# Surgical indication and management of obstructive colonic metastasis from primary lung adenocarcinoma: report of a case and review of the literature

**DOI:** 10.1186/s40792-024-02016-3

**Published:** 2024-09-18

**Authors:** Mai Watanabe, Shingo Tsujinaka, Tomoya Miura, Yoshihiro Sato, Yoh Kitamura, Kentaro Sawada, Atsushi Mitamura, Hiroto Sakurai, Noriko Kondo, Kazuhiro Takami, Kuniharu Yamamoto, Toru Nakano, Yu Katayose, Naruo Yoshimura, Chikashi Shibata

**Affiliations:** 1 Division of Gastroenterological and Hepato-Biliary-Pancreatic Surgery, Department of Surgery, Tohoku Medical and Pharmaceutical University, 1-15-1, Fukumuro, Miyagino, Sendai, Miyagi 983-8536 Japan; 2 Department of Respirology, Tohoku Medical and Pharmaceutical University, 1-15-1, Fukumuro, Miyagino, Sendai, Miyagi 983-8536 Japan

**Keywords:** Chemotherapy, Colonic metastasis, Gastrointestinal metastasis, Immunotherapy, Irradiation, Lung adenocarcinoma, Metastatectomy, Surgical resection

## Abstract

**Background:**

Colonic metastasis from lung cancer is very rare and is typically associated with poor prognosis. Herein, we report the case of a patient who achieved intermediate-term survival using a multimodal treatment approach, including chemotherapy, immunotherapy, radiotherapy, and surgical resection for obstructive colonic metastasis from primary lung adenocarcinoma.

**Case presentation:**

A woman in her 50s presented with anemia and a positive fecal occult blood test. Computed tomography revealed a tumor in the right upper lobe of the lung with mediastinal lymphadenopathy and wall thickening in the transverse colon. Colonoscopy revealed a stricture involving 50% of the colonic lumen. Biopsy revealed a poorly differentiated adenocarcinoma positive for CK-7 and TTF-1, very focally positive for napsin A, and negative for CK-20 and CDX-2. Furthermore, positron emission tomography/CT (PET/CT) showed a high maximum standardized uptake value (SUVmax) of 8.2 in the iliac bone. Based on these findings, the patient was diagnosed with primary lung adenocarcinoma with simultaneous metastasis to the transverse colon and iliac bone (cT4N3M1c, cStage IVB).

After receiving first-line chemotherapy with atezolizumab, pemetrexed, and carboplatin, the tumors shrank after 4 courses. Subsequently, the patient received maintenance therapy with atezolizumab and pemetrexed. However, the tumor enlarged after 10 courses. Second-line chemotherapy with docetaxel and ramucirumab (3 courses) failed to achieve tumor reduction. Colonoscopy revealed an impassable colonic tumor. Nineteen months after diagnosis, surgery was planned for imminent intestinal obstruction.

We determined that the colonic tumor was resectable, because laparoscopic exploration revealed no other metastases. The tumor was resected by partial colectomy with ileocolonic anastomosis. The postoperative course was uneventful. Pathological examination revealed a resection margin that was negative for malignancy, and the histological type was consistent with metastatic lung adenocarcinoma.

The patient then received nab-paclitaxel therapy; however, she developed symptoms of superior vena cava syndrome after 3 courses. The patient received palliative irradiation (30 Gy/10 fr) followed by nivolumab. She soon developed a solitary brain metastasis, and stereotactic irradiation was planned. After 3 courses of nivolumab, the metastasis was reduced significantly, and stereotactic brain irradiation was canceled. The lung tumor and mediastinal lymphadenopathy gradually shrank, and the patient survived for 13 months after surgery without disease progression.

**Conclusions:**

In this case, surgical resection of colonic metastasis from primary lung adenocarcinoma may have contributed to the short-term prognosis as a bridge-to-next available multimodal treatment.

## Background

The incidence and mortality rates of lung cancer have recently increased, particularly in developed countries compared to developing countries [1]. Approximately 50% of patients with lung cancer present with extrapulmonary metastasis, most commonly in the bone, liver, and brain [2, 3]. Gastrointestinal metastases are often asymptomatic and difficult to diagnose before death. The actual incidence rate varies (0.5–10%) and depends primarily on the evaluation method [4]. Colonic metastasis is a very rare type of gastrointestinal metastasis and is associated with a poorer prognosis compared to other gastrointestinal metastases [5]. Herein, we report the case of a patient who achieved intermediate-term survival with multimodal treatment, including chemotherapy, immunotherapy, and radiotherapy, for primary lung adenocarcinoma and surgical resection for obstructive colonic metastasis.

## Case presentation

**Fig. 1:**
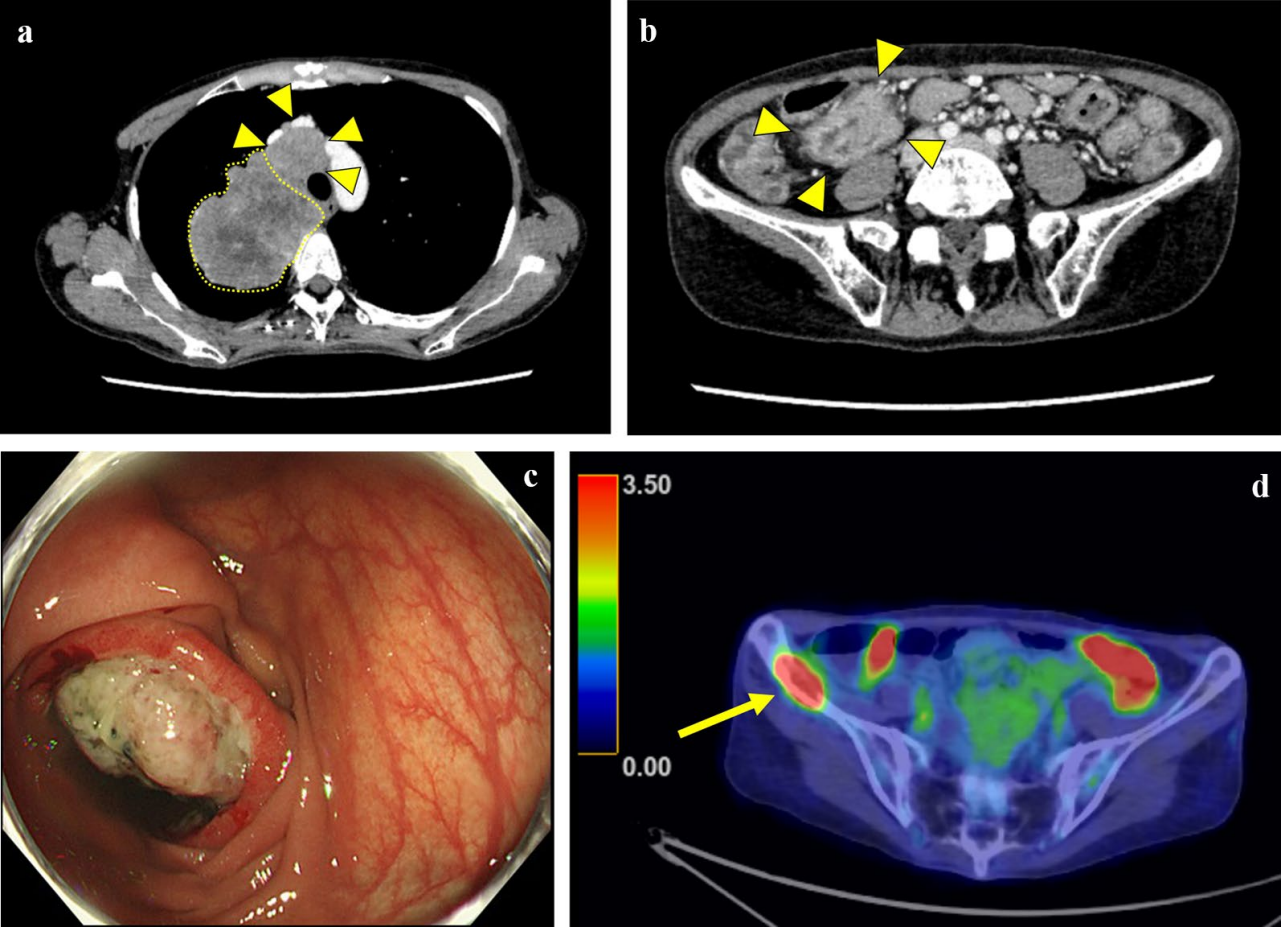
Imaging studies at the first diagnosis. **a** Chest computed tomography (CT) reveals a contrast-enhanced tumor exceeding 100 mm in diameter in the right upper lobe of the lung (traced with a dotted line) and adjacent mediastinal lymphadenopathy (arrowheads). **b** Abdominal CT reveals contrast-enhanced wall thickening in the transverse colon (arrowheads). **c** Colonoscopy shows an irregular, ulcerated, and raised lesion suggestive of an invasive tumor in the transverse colon. **d** Positron emission tomography/CT reveals increased ^18^F-fluorodeoxyglucose accumulation in the right iliac crest (arrow)

A woman in her 50s presented with anemia and a positive fecal occult blood test. Chest computed tomography (CT) revealed an irregular mass exceeding 100 mm in diameter in the right upper lobe of the lung with mediastinal lymphadenopathy (Fig. 1a). Contrast-enhanced CT also revealed wall thickening in the transverse colon near the hepatic flexure (Fig. 1b). Colonoscopy revealed a tumor located approximately half circumferentially in the hepatic flexure (Fig. 1c). Biopsy of the transverse colon tumor confirmed a poorly differentiated adenocarcinoma, which was positive for CK-7 and TTF-1, very focally positive for napsin A, but negative for CK-20 and CDX-2. Positron emission tomography/CT (PET/CT) revealed increased ^18^F-fluorodeoxyglucose accumulation with maximum standardized uptake values (SUVmax) of 16.1 in the right upper lobe of the lung, 9.3 in the transverse colon, and 8.2 in the right iliac bone (Fig. 1d).

Based on these findings, the patient was diagnosed with right upper lobe lung adenocarcinoma with simultaneous metastases to the transverse colon and iliac bone (cT4N3M1c, cStage IVB). The primary lung tumor was considered surgically unresectable. Initially, no surgical intervention or endoscopic stenting was planned for the colonic metastasis because of the lack of stenotic symptoms.

**Fig. 2:**
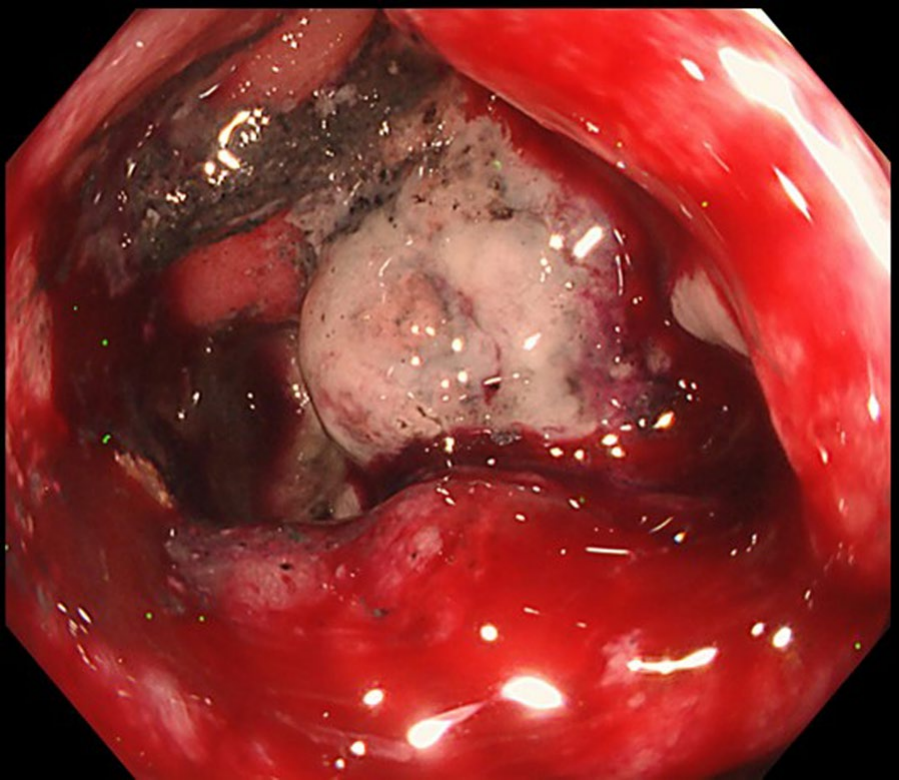
Follow-up colonoscopy after the second-line chemotherapy. Colonoscopy reveals a circumferential, endoscopically impassable tumor in the transverse colon

The patient initially received first-line chemotherapy comprising atezolizumab, pemetrexed, and carboplatin (PEM + CBCDA). The primary tumor in the right upper lobe had shrunk to 66 mm by the end of 4 courses. Subsequently, the patient was administered maintenance therapy with atezolizumab and pemetrexed. However, follow-up CT scans revealed tumor growth in the lung and colon after the completion of 10 courses. The treatment regimen was then modified to second-line chemotherapy comprising docetaxel and ramucirumab, and the primary and metastatic tumors enlarged again after 3 courses. Although the patient did not have any stenotic symptoms, a subsequent colonoscopy identified a fully circumferential obstructing tumor in the transverse colon (Fig. 2). Nineteen months after the lung cancer diagnosis, surgical intervention was planned for the colonic metastasis, considering imminent intestinal obstruction. To minimize surgical complications, we allowed a drug-free period of 9 weeks before surgery.

**Fig. 3:**
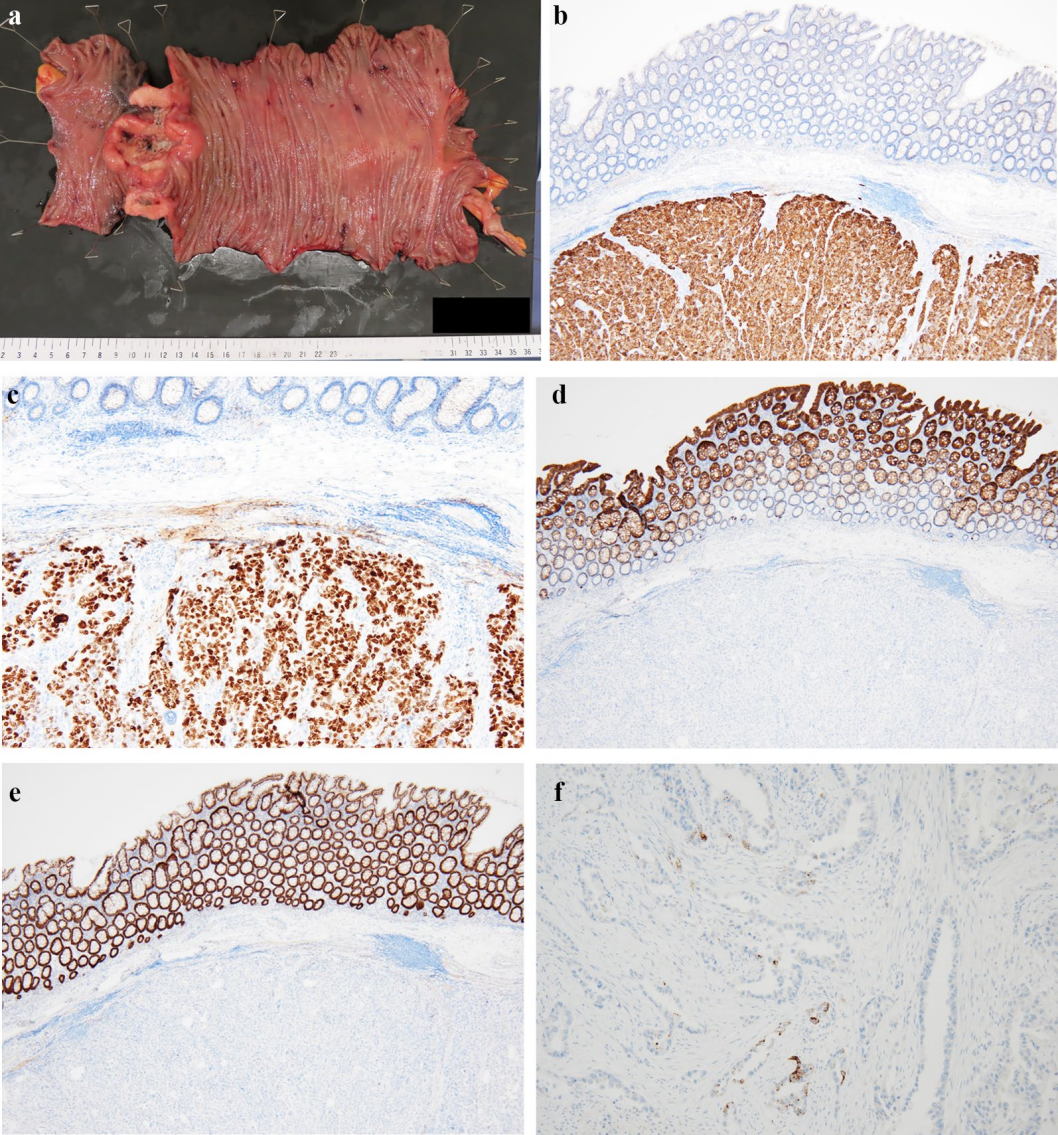
Pathological examination of the resected specimen. **a** Macroscopic view of the resected specimen (before formalin fixation). **b**–**f** Microscopic view with immunohistochemical staining. **b** CK-7 [magnification, × 40], **c** TTF-1 [magnification, × 40], **d** CK-20 [magnification, × 40], **e** CDX-2 [magnification, × 40], **f** napsin A [magnification, × 100]

Laparoscopic exploration revealed no peritoneal carcinomatosis or additional metastases beyond the colonic tumor. Further, the tumor demonstrated good mobility, suggesting no invasion of the surrounding organs, such as the pancreas or duodenum. Therefore, the tumor was intraoperatively determined to be completely resectable. Considering these factors and the tumor size, the laparoscopic approach was converted to open laparotomy for better access, and the wound was extended through a midline incision. The tumor was resected by partial colectomy with minimal lymph node dissection, and a 5-cm resection margin from the tumor was obtained. Furthermore, ileocolonic anastomosis was performed as a functional end-to-end anastomosis. The postoperative course was uneventful, and the patient was discharged 10 days after surgery. The macroscopic view of the resected specimen revealed an 80 × 60 mm, ulcerated, circumferential, and full-thickness tumor (Fig. 3a). The resection margins were negative for malignancy. Furthermore, the histological tumor type was moderately to poorly differentiated adenocarcinoma positive for CK-7 and TTF-1, very focally positive for napsin A, but negative for CK-20 and CDX-2 (Fig. 3b–f). These findings were identical to those of the initial colonic biopsy diagnosis.

**Fig. 4:**
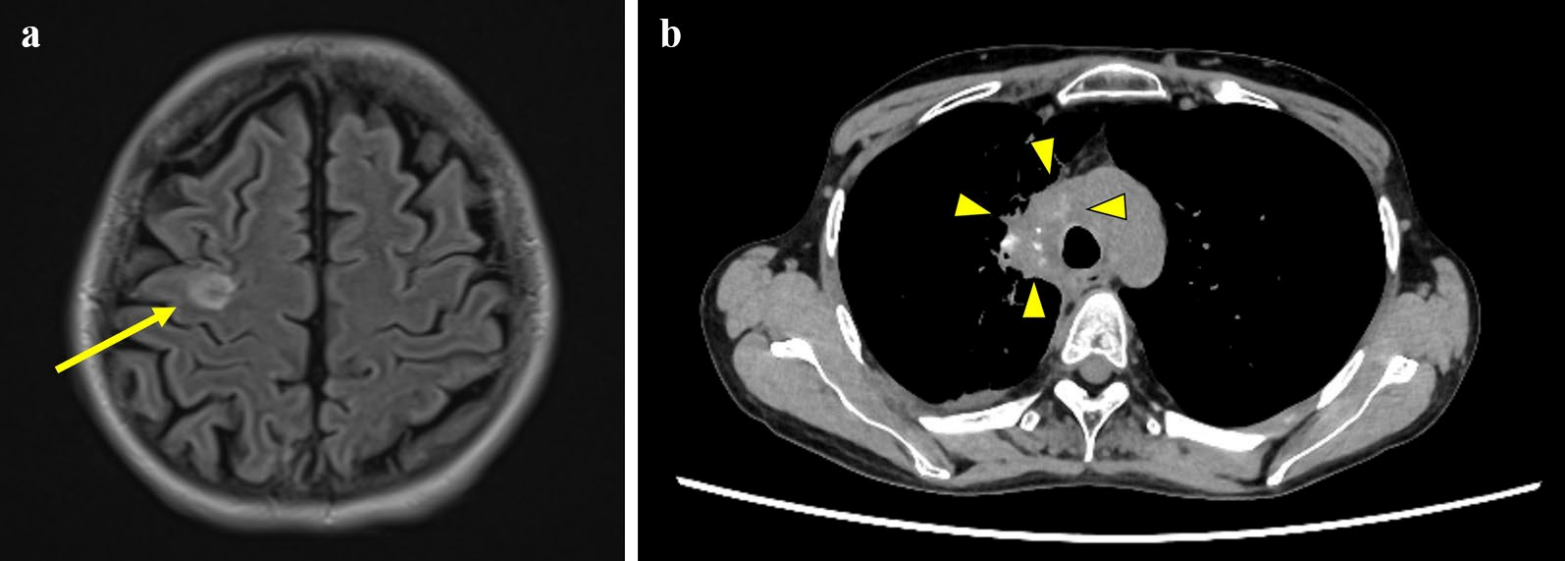
Follow-up imaging studies after surgery. **a** Brain MRI with FLAIR/T2WI (fluid attenuated inversion recovery/T2-weighted image) shows solitary metastasis in the right frontal lobe (arrow). **b** Chest computed tomography shows shrinkage of the primary lung tumor and mediastinal lymphadenopathy with an ill-defined boundary (arrowheads)

One month after surgery, the patient received nab-paclitaxel treatment. However, after 3 courses, she developed superior vena cava syndrome caused by a progressive lung tumor with mediastinal lymphadenopathy. Palliative irradiation therapy (at a dose of 30 Gy/10 fr) was then administered. Following irradiation, the patient received underwent immunotherapy with nivolumab. Soon after, a solitary brain metastasis was diagnosed (Fig. 4a), requiring stereotactic irradiation. After 3 courses of nivolumab, the brain metastasis was reduced significantly, and stereotactic irradiation was canceled. Additionally, the primary tumor and mediastinal lymphadenopathy gradually shrank (Fig. 4b). No recurrence of colonic metastasis was observed, and the iliac bone showed only sclerotic changes. The patient remained alive without disease progression at the end of 7 nivolumab courses (13 months after surgery).

## Discussion

Surgical intervention for extrathoracic metastases is typically based on resectability; with aggressive metastatectomies uncommon due to patients often being asymptomatic. Most surgical interventions for colonic metastasis occur in patients experiencing stenotic symptoms, such as nausea, vomiting, and abdominal pain caused by intestinal obstruction [6, 7]. A literature search using PubMed/MEDLINE (1996–2024) showed that 17 patients (reported in 15 reports) survived for at least 6 months after diagnosis or elective surgical resection of colorectal metastases from lung cancer (Table 1) [8–22]. The mean survival time for these patients was 14 months, exceeding that reported in previous studies [5, 23]. Patients with extracolonic metastases have a poorer prognosis than those without extracolonic metastases, although successful treatment with simultaneous colonic resection has been reported for cases with other gastrointestinal metastases (Table 1) [10, 18]. Surgery for gastrointestinal metastases aims to prevent obstruction or perforation and improve palliative care, quality of life, and short-term survival when the primary tumor or other metastatic lesions are oncologically well-controlled [24–27].

**Table 1:** Summary of previously reported patients with colorectal metastases from primary lung cancer

Author (year), [ref.]	Age, sex	Histology	Region of colon metastases	Sites of metastases except colon	Surgical procedure	Non-surgical treatment and timing	Use of molecular targets or immune checkpoint inhibitors	Survival time (months)	Survival status
Carr CS, et al. (1996) [17]	60, F	Scc	Transverse colon	None	Extended right hemicolectomy	None	None	24**	Dead
Carr CS, et al. (1996) [17]	52, F	Scc	Transverse colon	None	Extended left hemicolectomy	None	None	24**	Alive
Rouhanimanesh Y, et al. (2001) [10]	56, M	Scc	Sigmoid colon	Jejunum, bladder	Sigmoid colectomy, partial small bowel resection	None	None	13**	Alive
Carroll D, et al. (2001) [12]	68, M	Scc	Sigmoid colon	None	Sigmoid colectomy	Chemotherapy after surgery	None	8**	Dead
Uner A, et al. (2005) [13]	58, M	Scc	Descending colon	None	Left hemicolectomy	None	None	9**	Alive
Ono H, et al. (2009) [8]	59, M	Adeno	Descending colon	Brain	Left hemicolectomy	Chemotherapy and stereotactic radiotherapy after surgery	None	12*	Alive
Lee PC, et al. (2011) [9]	61, F	Adeno	Unknown	None	Right hemicolectomy	Chemotherapy before surgery	Unknown	8*	Alive
Lee PC, et al. (2011) [9]	66, M	Scc	Unknown	Liver brain	Right hemicolectomy	Unknown	Unknown	5*	Dead
Lee PC, et al. (2011) [9]	54, F	Adeno	Unknown	Bone brain	Right hemicolectomy	Unknown	Unknown	4*	Dead
Fujiwara A, et al. (2011) [11]	66, F	Pleo	Unknown	None	Unknown	Unknown	Unknown	40**	Alive
Fujiwara A, et al. (2011) [11]	83, M	Pleo	Unknown	Liver	Unknown	Unknown	Unknown	3.7**	Dead
Sakai H, et al. (2012) [15]	60, F	Scc	Sigmoid colon	None	Sigmoid colectomy, partial transverse colectomy	Chemoradiotherapy before surgery, chemotherapy after surgery	None	6**	Alive
Doussot A, et al. (2013) [19]	62, M	Adeno	Ascending colon	None	Right hemicolectomy	Chemotherapy after surgery	None	6**	Dead
Vittorakis S, et al. (2018) [14]	49, M	Adeno	Ascending colon	None	Right hemicolectomy	Chemotherapy after surgery	None	12**	Alive
Suzuki Y, et al. (2019) [16]	79, F	Adeno	Rectum	Hilar and mediastinal lymph node	Low anterior resection	Multiple lines of chemotherapy with immunotherapy before surgery, chemotherapy after surgery	Gefitinib, bevacizumab, erlotinib, nivolumab, and afatinib	6**	Alive
Wang R, et al. (2019) [22]	47, F	Adeno	Sigmoid colon	None	Sigmoid colectomy	Chemotherapy before surgery, immunotherapy after surgery	Gefitinib	8**	Alive
Prabhakaran S, et al. (2020) [21]	85, M	Adeno	Ascending colon	None	Right hemicolectomy	None	None	24**	Alive
Catalano M, et al. (2022) [18]	78, M	Adeno	Transverse colon	Stomach and abdominal lymph node	Partial colectomy total gastrectomy	Chemotherapy (1st line) and immunotherapy (2nd line) after surgery	Pembrolizumab	48**	Alive
Nakayama Y, et al. (2023) [20]	83, M	Scc	Descending colon	None	Left hemicolectomy	Chemoradiotherapy with immunotherapy before surgery	Durvalumab	13*	Alive

*M* male, *F* female, *Scc* squamous cell carcinoma, *Adeno* adenocarcinoma, *Pleo* pleomorphic carcinoma, *U* unknown

^*^Survival time after diagnosis of gastrointestinal metastases

^**^Survival time after surgery for gastrointestinal metastases

Approximately 70.5% of gastrointestinal metastases from primary lung cancer involve multiple sites [5]. Therefore, a thorough evaluation is necessary to identify any additional distant metastases before surgery for patients with gastrointestinal metastases. Even in cases with multiple small intestinal or colonic metastases, surgical resection may be indicated for localized lesions unless the surgery is overly invasive and requires multivisceral resection with complex gastrointestinal reconstruction. For patients who cannot tolerate radical surgery, stoma creation or endoscopic stenting may be considered as palliative options [28]. In the present case, surgical intervention was undertaken to prevent intestinal obstruction, confirming tumor localization and resectability and preserving options for chemotherapy. Endoscopic colonic stenting or stoma creation offers a less invasive option. However, future therapeutic agents may include anti-vascular endothelial growth factors, such as bevacizumab or ramucirumab, which could increase the risk of perforation of the remaining tumor. Stoma creation can be easily performed and allows for prompt induction and continuation of the next treatment. However, in this case, the appropriate stoma site would have been the small intestine, which may have decreased tolerance to subsequent chemotherapy. The patient in the present study was judged tolerable to full surgical options because of the younger age and lack of significant comorbidities. The risks, benefits, advantages, and disadvantages of each interventional option were meticulously discussed, and the patient wished to undergo resection and anastomosis. After that, surgery was performed after obtaining a sufficient drug-free period.

TTF-1 and napsin A typically show high sensitivity and specificity for primary and metastatic lung adenocarcinomas [29]. A previous study showed that 79.2% of lung primary adenocarcinomas showed a napsin A + /TTF-1 + double-positive immunostaining pattern. In contrast, TTF-1^−^/napsin A + , TTF-1 + /napsin A^−^, and TTF-1^−^/napsin A^−^ were seen in 8.3%, 3.3%, and 9.2% of lung primary adenocarcinomas, respectively [30]. These results indicate that a few groups of lung adenocarcinomas may show “TTF-1 + /napsin A^−^” pattern in immunohistochemical staining. In this patient, positivity for napsin A was weak (Fig. 3f), while positivity for TTF-1 was strong (Fig. 3c), which suggests that the lung is the primary site of the cancer. Furthermore, negativity for CK-20 (Fig. 3d) and CDX-2 (Fig. 3e) and positivity for CK-7 (Fig. 3b) were incompatible with a gastrointestinal/colorectal primary site of origin [31, 32]. Considering these pathological and radiological findings, the patient was diagnosed with colonic metastasis from primary lung adenocarcinoma.

Recent real-world data suggest that tyrosine kinase inhibitors and immune checkpoint inhibitors may improve overall survival in patients with non-small cell lung cancer [33]. In the present case, the patient benefited from nivolumab therapy with good tumor control for primary lung cancer with mediastinal lymphadenopathy following complete resection of uncontrollable colonic metastasis. Therefore, local control of metastatic lesions is becoming increasingly important in lung cancer treatment.

This case report has some limitations. The diagnosis of primary lung cancer was based on radiological findings without cytology or biopsy results directly obtained from the lung tumor. In this case, the metastatic colonic tumor did not recur after surgical resection; however, the primary tumor had grown, and a new extrathoracic metastasis had occurred in the brain. Additionally, the molecular profiles and drug sensitivities may differ between primary and metastatic lesions. The literature review included only elective surgical cases that achieved reasonable survival times and did not include those that underwent emergency surgery with poorer survival, in whom surgery was inevitable and its indication was unquestionable.

## Conclusions

In this case, surgical resection of colonic metastasis from primary lung adenocarcinoma may have contributed to the short-term prognosis as a bridge-to-next available multimodal treatment.

## Data Availability

The datasets supporting the findings and inferences of this case report are included in this article.

## Abbreviations

CT: Computed tomography
PET: Positron emission tomography
PEM + CBCDA: Atezolizumab, pemetrexed, carboplatin
